# LncRNA-AK149641 regulates the secretion of tumor necrosis factor-α in P815 mast cells by targeting the nuclear factor-kappa B signaling pathway

**DOI:** 10.1038/s41598-020-73186-x

**Published:** 2020-10-06

**Authors:** Yao Zhou, Li-na Gu, Jie Zhang, Jing Pan, Jia-min Zhang, De-yu Zhao, Feng Liu

**Affiliations:** 1 Department of Respiratory Medicine, Children’s Hospital of Nanjing Medical University, No 72 Guang Zhou Road, Nanjing, 210008 Jiangsu China; 2 The Affiliated Wuxi Children’s Hospital of Nanjing Medical University, Wuxi, 214000 Jiangsu China; 3 Department of Emergency Medicine, Children’s Hospital of Nanjing Medical University, Nanjing, 210008 Jiangsu China

**Keywords:** Nuclear receptors, Long non-coding RNAs

## Abstract

Long noncoding RNAs play important roles in various biological processes. However, not much is known about their roles in inflammatory response. Mast cells, involved in innate and adaptive immunity, are one of the major effector cells in allergic inflammatory reactions and contribute to the pathogenesis of disorders, including asthma. In the present study, we aimed to verify and elucidate the function and possible role of a novel lncRNA, called lncRNA-AK149641, in the mechanism of lipopolysaccharide (LPS)-induced inflammatory response in P815 mast cells. The results showed that downregulating lncRNA-AK149641 decreased secretion of tumor necrosis factor-α into the supernatants of LPS-stimulated mast cells. Mechanistically, the activity of nuclear factor-kappa B (NF-κB) decreased after downregulating lncRNA-AK149641, as shown by western blot and electrophoretic mobility shift assays. Moreover, RNA binding protein immunoprecipitation (RIP) verified that lncRNA-AK149641 was able to bind to NF-κB in the nucleus. In conclusion, we demonstrated that lncRNA-AK149641 regulated LPS-induced inflammatory response in mast cells through the NF-κB signaling pathway.

## Introduction

As resident tissue cells, mast cells are distributed particularly near the surfaces exposed to the environment, such as respiratory system. They secrete various cytokines and chemokines, and thus, are involved in many biological processes, including inflammatory and immune responses. As resident inflammatory cells of airways, mast cells are recognized as key elements in the pathogenesis of asthma^1–3^. Localization within the airway smooth muscle bundle affects the development of airway hyperresponsiveness^4^.

Mast cells can be activated by Toll like receptors (TLRs)^5^, which are the principal pattern recognition receptors in the innate immune system^6^, through stimulation of LPS^7^. NF-κB is located downstream of the TLR signaling pathway, and is pivotal to regulate the immune and inflammatory response^8^. Besides, myeloid differentiation primitive-response protein 88 (MyD88) is an important molecule in the LPS-induced canonical NF-κB signaling pathway^9,10^. By the way, when exposed to sensitizing allergen, mast cells degranulate and release biologically active products to take part in inflammatory reaction^11^.

LncRNAs consist of more than 200 nucleotides and do not possess protein-coding capability. They participate in various biological and pathological processes, such as cancer^12,13^, metabolism^14^, and immune responses^15,16^. To date, several studies have demonstrated that some lncRNAs are involved in inflammatory responses. For example, lncRNA-Cox2 regulates intestinal epithelial inflammatory responses by modulating interleukin (IL)-12b transcription^17^.

In this study, we aimed to identify potential novel lncRNAs related to inflammatory responses and elucidate their roles in the mechanism of LPS-mediated inflammation. We performed microarray assays to screen differentially expressed lncRNAs in LPS-stimulated P815 mast cells and normal control mast cells, and the identified lncRNAs were further studied. After downregulating candidate lncRNAs in P815 mast cells, concentration of TNF-α was significantly reduced in supernatants from cells in which lncRNA-AK149641 was downregulated. To further investigate its function and possible role in the mechanism of LPS-mediated inflammatory responses, EMSA and RIP were performed.

## Results

### Differentially expressed lncRNAs between LPS-stimulated and normal control P815 mast cells

**Figure 1:**
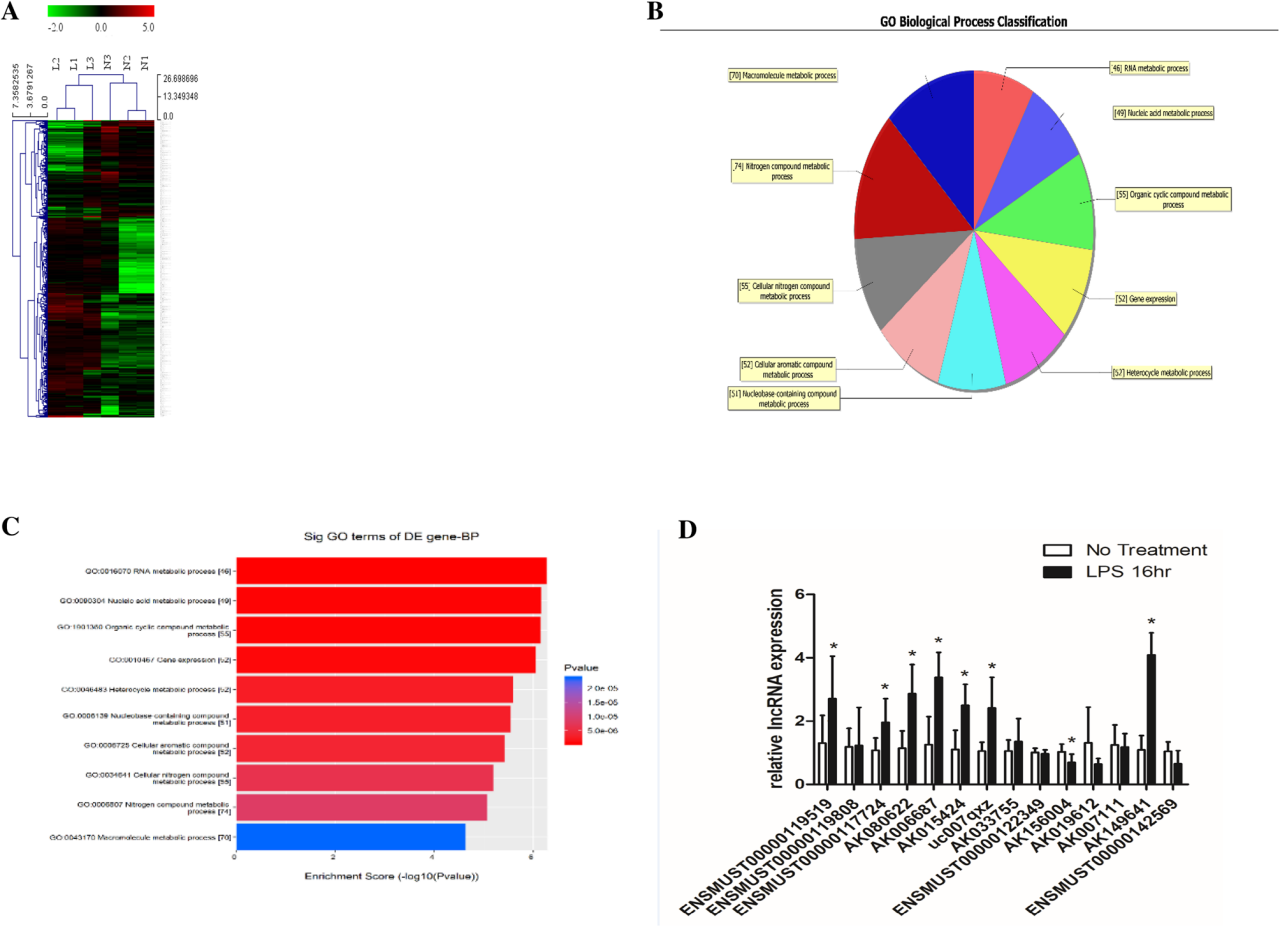
Differentially expressed lncRNAs between LPS-stimulated and normal control P815 mast cells. (**A**) Heat map of lncRNAs expressed in LPS-stimulated and normal control cells. (**B**–**C**) Gene ontology analysis. (**D**) Expressions of 14 candidate lncRNAs in P815 mast cells upon stimulation with LPS. (N1, N2, and N3: normal control P815 mast cells; L1, L2, and L3: LPS-stimulated P815 mast cells) (*P < 0.05, n = 3).

To identify lncRNAs that may be involved in LPS-stimulated inflammatory responses, microarray assays were performed on LPS-stimulated and normal control P815 mast cells to reveal differentially expressed lncRNAs (Fig. 1A). A gene ontology bioinformatics analysis was also performed (Fig. 1B,C). Based on the microarray results, 14 significantly upregulated candidate lncRNAs were selected for further experiments. The quantitative real-time polymerase chain reaction (qRT-PCR) analysis in LPS-stimulated P815 mast cells showed a similar expression pattern (Fig. 1D) to the microarray analysis. Seven lncRNAs were upregulated upon LPS stimulation, among which, lncRNA-AK149641 showed the most obvious increase.

### Downregulation of lncRNA-AK149641 reduced concentration of TNF-α in supernatants of mast cells

**Figure 2:**
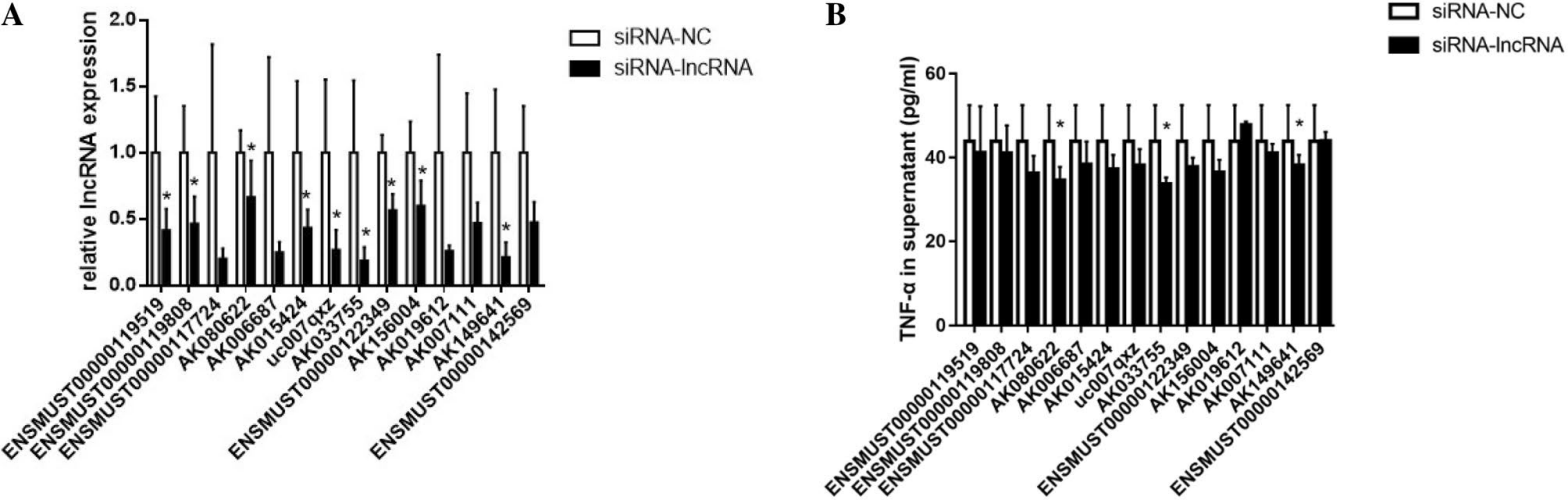
LncRNA-AK149641 regulates TNF-α secretion in P815 mast cells. (**A**) Expressions of 14 candidate lncRNAs are significantly decreased by siRNA treatment. (**B**) Concentration of TNF-α in the supernatants (*P < 0.05, n = 3).

To determine whether any of the 14 differentially expressed lncRNAs participated in the inflammatory response, P815 mast cells were transfected with three different small interfering (si) RNAs for each candidate lncRNA to efficiently downregulate expressions. Downregulation was verified by qRT-PCR (Fig. 2A). Concentrations of proinflammatory cytokine TNF-α in the supernatants were measured by Enzyme-Linked ImmuoSorbent Assay (ELISA). The result showed that downregulation of certain lncRNAs, that is, lncRNA-AK080622, lncRNA-AK033755 and lncRNA-AK149641, modulated the secretion of TNF-α (Fig. 2B). Based on Fig. 1D, upon stimulation of LPS, lncRNA-AK033755 was not upregulated, we first excluded it. Considered that the level of TNF-α in supernatants is similar between downregulation of lncRNA-AK080622 and lncRNA-AK149641, however, the expression of lncRNA-AK149641 was extremely upregulated in LPS stimulated P815 mast cells, we finally selected lncRNA-AK149641 for further study.

### LncRNA-AK149641 participated in the LPS-induced inflammatory response

**Figure 3:**
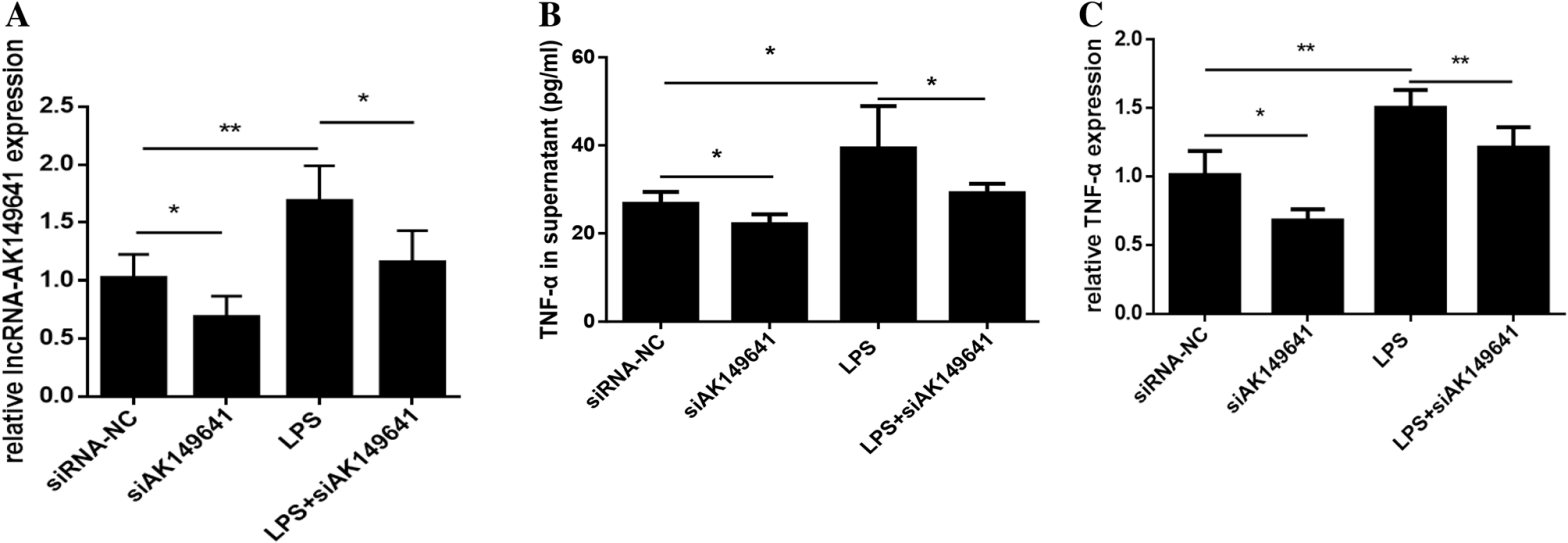
Effect of lncRNA-AK149641 on the LPS-induced secretion of TNF-α. (**A**) Effects of LPS stimulation and siRNA transfection on the expression of lncRNA-AK149641 in P815 mast cells. Effects of LPS stimulation and siRNA transfection on the secretion of TNF-α, determined by (**B**) ELISA and (**C**) qRT-PCR (*P < 0.05, **P < 0.01, n = 6).

To explore whether lncRNA-AK149641 was involved in the secretion of TNF-α, P815 mast cells were divided into four groups: negative control (siRNA-NC), lncRNA-AK149641 downregulated (transfected with siRNA targeting lncRNA-AK149641; siAK149641), LPS stimulation (LPS), LPS stimulation with lncRNA-AK149641 downregulated (LPS + siAK149641). Following transfection with three different siRNAs, mast cells were cultured for 24 h and supernatants were collected for detecting concentration of TNF-α. The results showed that stimulation with LPS increased the expression of lncRNA-AK149641 while transfection of siRNA efficiently downregulated the expressed level (Fig. 3A). Moreover, downregulating lncRNA-AK149641 decreased the secretion of TNF-α determined both by ELISA (Fig. 3B) and qRT-PCR (Fig. 3C). These results indicated that lncRNA-AK149641 may be a regulatory factor in the LPS-stimulated inflammatory response, including the secretion of TNF-α.

### Subcellular localization of lncRNA-AK149641 in P815 mast cells

**Figure 4:**
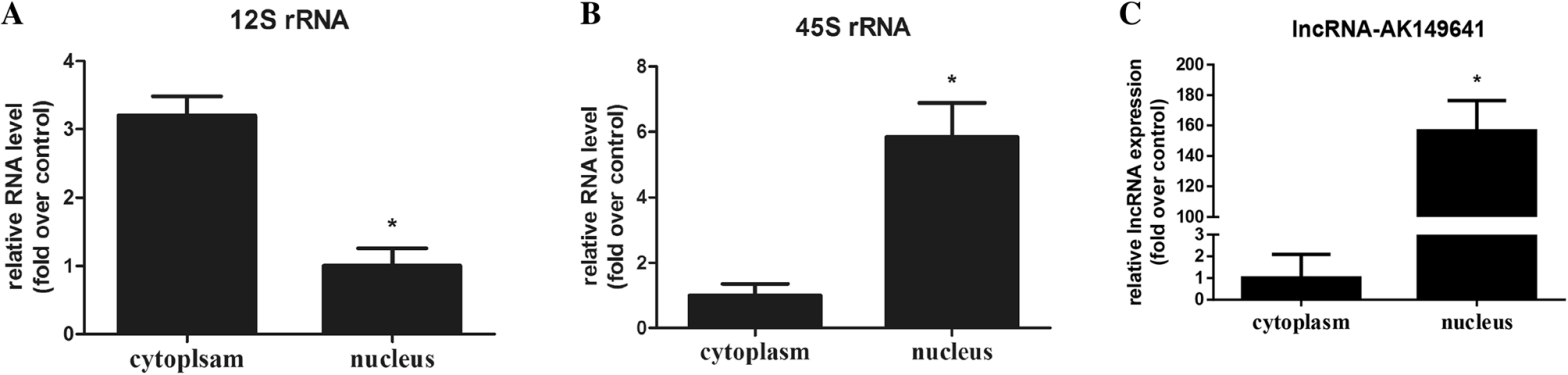
Expression levels of 12S rRNA, 45S rRNA, and lncRNA-AK149641 in the nucleus and cytoplasm of P815 mast cells (*P < 0.05, n = 3).

To explore the subcellular localization of lncRNA-AK149641 in P815 mast cells, we extracted cellular RNAs from nuclei and cytoplasm, and then measured the expression of lncRNA-AK149641 in these fractions. The results showed that lncRNA-AK149641 was expressed at a significantly higher level in the nucleus than that in the cytoplasm (Fig. 4).

### NF-κB activity decreased in lncRNA-AK149641 downregulated P815 mast cells

**Figure 5:**
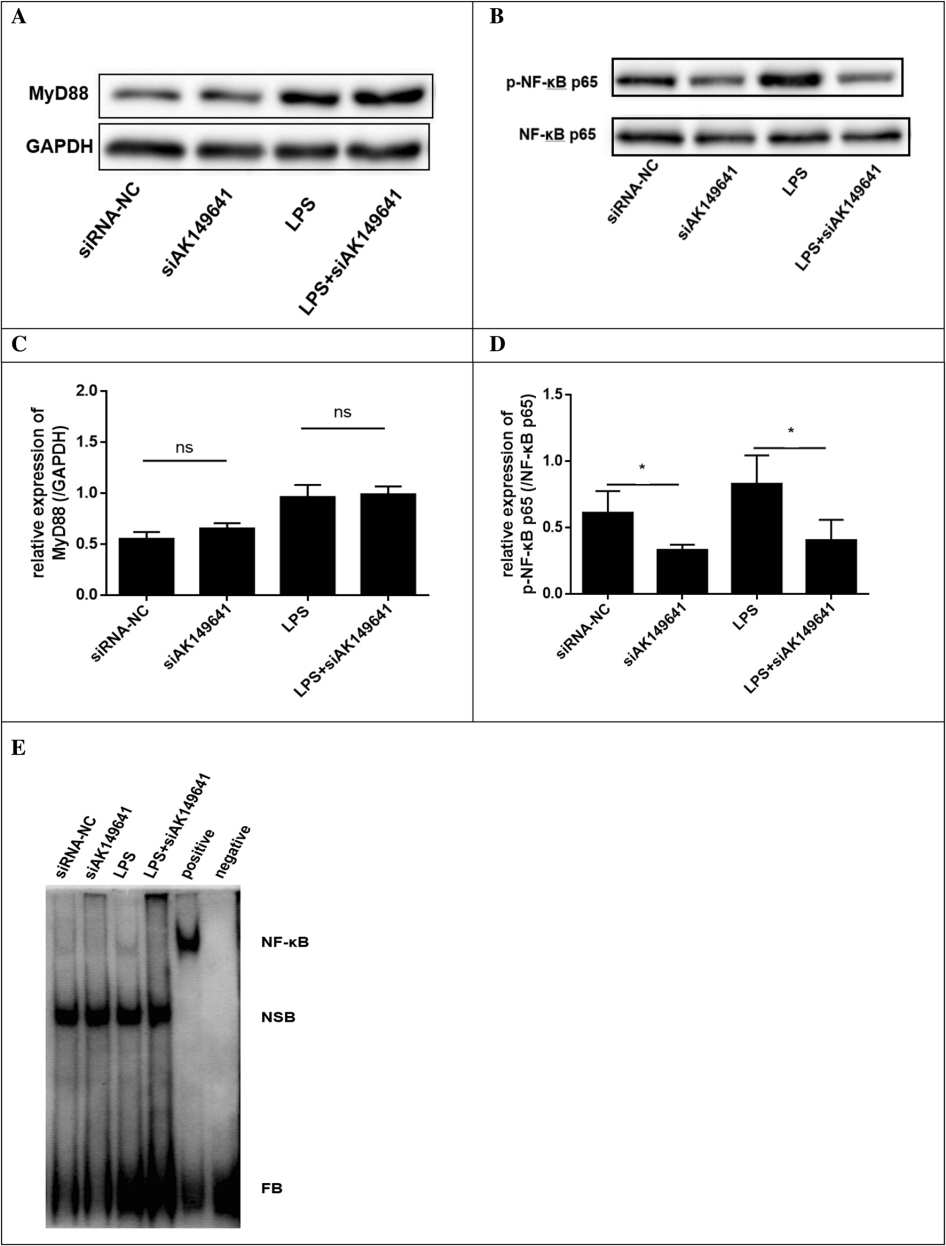
Changes in NF-κB expression in response to different lncRNA-AK149641 levels. (**A**–**D**) Western blots showing the expression of the MyD88 and p-NF-κB p65 proteins. p-NF-κB p65 decreased in the lncRNA-AK149641 downregulated group after LPS stimulation. There was no significant difference in the expression of MyD88. (**E**) The EMSA shows that downregulation of lncRNA-AK149641 inhibits the expression of NF-κB (NSB: Non-Specific Binding; FB: Free Biotin Labeled Probe) (*P < 0.05, n = 3).

To better understand the relationship between lncRNA-AK149641 and the NF-κB signaling pathway in LPS-induced inflammatory responses, the expressions of MyD88 and phosphorylated (p)-NF-κB p65 were examined by western blot analysis. We also examined NF-κB proteins by EMSA, using nuclear extracts from cells with downregulated lncRNA-AK149641. As shown in Fig. 5A,B,C,D, expression of p-NF-κB P65 decreased in the lncRNA-AK149641 downregulated group after LPS stimulation, compared to the negative control group. In contrast, there was no significant difference in the expression of MyD88. NF-κB DNA binding in the nuclear fraction, examined by EMSA, was lower in cells with downregulated lncRNA-AK149641 compared with negative control cells (Fig. 5E). These results indicated that lncRNA-AK149641 regulated the LPS-induced inflammatory response in mast cells through the NF-κB signaling pathway.

### LncRNA-AK149641 bound to NF-κB in the nucleus

**Figure 6:**
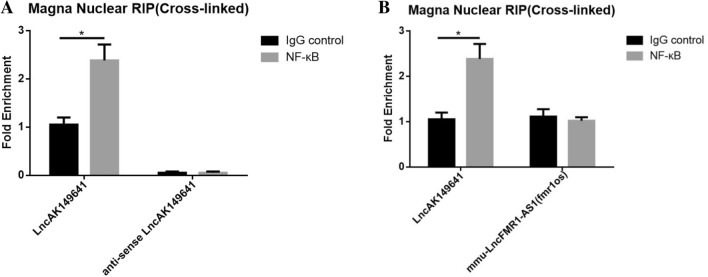
LncRNA-AK149641 bound to NF-κB in the nucleus. Whole-cell lysates were prepared and incubated with either isotype IgG control (isotype) or NF-κB antibodies. Immunoprecipitates were harvested with protein A/G Sepharose beads. RNA was isolated and the lncRNA transcripts in the immunoprecipitates were determined by the qRT-PCR. (**A**) LncRNA-AK149641 and anti-sense lncRNA-AK149641. (**B**) LncRNA-AK149641 and mmu-lncRNA FMR1-AS1. (*P < 0.05, n = 4).

RIP was performed to verify the relationship between lncRNA-AK149641 and the NF-κB signaling pathway by using isotype IgG control and NF-κB antibodies. To identify that NF-κB binds to lncRNA-AK149641 specifically, we adopted both antisense primers of lncRNA-AK149641 (Fig. 6A) and mmu-lncRNA named FMR1-AS1 (Fig. 6B) as control. The analysis showed that lncRNA-AK149641 was able to bind to NF-κB specifically in the nucleus.

## Discussion

Non-coding RNAs is a general term for all RNAs that cannot be translated into proteins. Recently, various studies have shown that non-coding RNAs participate in the pathogenesis of several diseases by regulating the function of mast cells. For example, by affecting mast cell proliferation and apoptosis, miR-490-5p is involved in the pathogenesis of irritable bowel syndrome^18^. Moreover, reports have shown that some lncRNAs take part in LPS-induced biological processes. For instance, lincRNA-Cox2 regulates late-primary inflammatory response genes stimulated by LPS^19^. In cardiomyocytes of mice with LPS-induced sepsis, upregulation of lncRNA HOTAIR promotes TNF-α production by activating NF-κB, involving the p-NF-κB p65 subunit. In addition, silencing HOTAIR preserves cardiac function during LPS-induced sepsis^20^.

The current study was designed to identify how lncRNA-AK149641 regulates the inflammatory response. TNF-α is an important cytokine in triggering and sustaining inflammation in mast cells^21^. The results showed that, by downregulating lncRNA-AK149641 in LPS-stimulated P815 mast cells, the secretion of TNF-α decreased. Together with the results of previous studies, we concluded that lncRNA-AK149641 regulated TNF-α secretion in mast cells.

Recent studies have demonstrated that lncRNAs and cytokines can regulate each other. For example, the lncRNA THRIL regulates the expression of TNF-α through its interaction with heterogeneous ribonucleoproteins^22^. LincRNA-p21, a negative regulator of TNF-α-stimulated NF-κB activity, reduces the inflammatory response in rheumatoid arthritis^23^. In response to stimulation by TNF-α, lincRNA-Cox2 suppresses transcription of the IL-12b gene by recruiting the Mi-2/NuRD repressor complex, thus regulating intestinal epithelial inflammatory responses^17^. Additionally, lncRNA Lethe can be induced selectively by TNF-α through the NF-κB signaling pathway and, in turn, regulates the secretion of TNF-α through negative feedback^24^. Furthermore, TNF-α upregulates the expression of inducible co-stimulatory molecule ligand on mast cells and inhibits degranulation, thus promoting the differentiation of regulatory T cells and inducing a shift in cytokine expression from a Th1 to a Th2 profile.

In our study, expression of lncRNA-AK149641, and concentrations of TNF-α in supernatants from P815 mast cells, were upregulated by LPS-stimulation. Importantly, downregulating lncRNA-AK149641 attenuated these effects significantly. Collectively, this information suggests that lncRNA-AK149641 may be a regulator of the LPS-induced inflammatory response and the release of proinflammatory cytokines.

A lot of studies have shown that TLRs are important signal receptors and are involved in the secretory activity of mast cells after LPS stimulation. NF-κB is an important transcription factor downstream of TLR pathway. One study showed that knockdown of nuclear-localized, NF-κB-regulated, eRNAs (IL-1b-eRNA), and RBT (IL-1b-RBT46) surrounding the IL-1b locus, attenuated LPS-induced mRNA transcription and release of the pro-inflammatory mediators, IL-1b, and CXCL8, thus regulating the immune response^25^.

Based on these findings, we focused on the NF-κB signaling pathway. Our data showed that, after downregulating lncRNA-AK149641, the level of MyD88 was not significantly changed, but the expression of p-NF-κB p65 was significantly lower than that of negative control cells. The EMSA also showed that NF-κB DNA binding in the nuclear fraction was lower in lncRNA-AK149641 downregulated mast cells. Furthermore, RIP revealed that lncRNA-AK149641 was able to bind to NF-κB in the nucleus.

In summary, these data demonstrate that lncRNA-AK149641 is involved in LPS-induced secretion of TNF-α in mast cells probably through the NF-κB signaling pathway.

## Materials and methods

### Cell lines and culture

The mouse mastocytoma cell line, P815, was purchased from the Cell bank of the Chinese Academy of Sciences (Shanghai, China). P815 mast cells were cultured in Dulbecco’s modified Eagle’s medium containing 4.5 g/L glucose, and supplemented with 10% fetal bovine serum (WISENT, Nanjing, China), 100 U/ml penicillin, and 100 µg/ml streptomycin in humidified air at 37˚C with 5% CO_2_. Cells were cultured at a density of 1.5 × 10^6^ cells/ml and treated with or without 1 µg/ml LPS (Sigma-Aldrich, St. Louis, MO, USA). After 16 h, cultured cells were washed twice with PBS and then collected.

### LncRNA microarray assay

Triplicate RNA samples extracted from LPS-stimulated and untreated P815 mast cells were used for lncRNA microarray assays. Microarray experiments were performed by Kangchen Bio-tech (Shanghai, China). The thresholds set to identify upregulated or downregulated genes were fold changes of ≥ 2.0 or ≤ 0.05, respectively.

### RNA interference

Three different siRNAs that targeted one lncRNA, and a scrambled siRNA control, were designed and synthesized by GenePharma (Shanghai, China). The siRNA molecules were 20-bp doubled-stranded oligonucleotides with proprietary chemical modifications. The sequences of these siRNAs are summarized in Supplementary Table 1.

### Transfection of P815 cells

P815 mast cells cultured on six-well plates were transfected with siRNA or a scrambled siRNA control using Lipofectamine 2000 (Invitrogen, Carlsbad, CA, USA) according to the manufacturer’s instructions. Cells were harvested after 24 h for performing qRT-PCR and other experiments.

### Total RNA extraction and qRT-PCR analysis

Total RNA was extracted from cultured cells with TRIzol reagent (Invitrogen) according to the manufacturer’s instructions. Total RNA (1000 ng) was reverse transcribed in a final volume of 20 μl using the RevertAid First Strand cDNA Synthesis Kit (Thermo Fisher Scientific, Waltham, MA, USA). qRT-PCR was performed using the FastStart Universal SYBR Green Master Mix (Roche, Basel, Switzerland) on an ABI 7500 system (Applied Biosystems, Foster City, CA, USA) according to the manufacturer’s instructions. The expression level of lncRNAs was normalized to that of GAPDH using the $2^{{ - \Delta \Delta {\text{CT}}}}$ method. The primer sequences used for lncRNA amplification are summarized in Supplementary Table 2.

### ELISAs

Culture supernatants were collected 24 h after transfection and stored at − 80 °C. The concentration of TNF-α was measured by ELISA kits (R&D Systems, Minneapolis, MN, USA) according to the manufacturer’s instructions.

### Nuclei and cytoplasm extraction

The nuclei and cytoplasm of P815 cells were extracted separately by using a PARIS kit (Thermo Fisher Scientific) according to the manufacturer’s instructions.

### Western blot analyses

Total protein was separated and transferred onto nitrocellulose membranes (EMD Millipore, Billerica, MA, USA). Membranes were blocked with 5% non-fat milk, then incubated overnight at 4 °C with specific antibodies. After washing with tris-buffered saline/0.1% Tween 20, the membranes were incubated with a horseradish peroxidase-conjugated anti-rabbit antibody at room temperature for 2 h. Signals were detected on a gel imaging system using an enhanced chemiluminescence western blotting substrate (Thermo Fisher Scientific).

### EMSA

NF-κB protein was extracted from the nuclei of cells (Vazyme, Piscataway, NJ, USA). The assay was performed using an EMSA kit (Pierce, Rockford, IL, USA) according to the manufacturer’s recommendations. The 5′-biotin-labeled NF-κB oligo (5′-AGTTGAGGGGACTTTCCCAGGC-3′) was purchased from Sigma-Aldrich.

### RIP

This assay was performed by a Magna Nuclear RIP (Cross-Linked) Kit (EMD Millipore) according to the manufacturer’s instructions. All buffers used contained an RNase inhibitor. Nuclei were isolated and used for chromatin fragmentation. After immunoprecipitation with IgG control and NF-κB antibodies, the beads were washed and RNA was eluted, and precipitated by ethanol. The precipitated RNA pellets were resuspended in nuclease-free water. An aliquot of RNA was used for the cDNA synthesis reaction and qRT-PCR analysis.

### Statistical analyses

Statistical analyses were performed with SPSS 20.0 software (IBM, Chicago, IL, USA). Comparisons between groups were applied using Student’s t test or multiple comparisons using one-way analysis of variance (ANOVA) test. Since analysis of variance was significant, comparisons were applied using the Dunnett’s test or Tukey’s test. Differences were considered statistically significant if the *P* value was < 0.05.

## Supplementary information

Supplementary Information.
