# Supplementary material for: LncRNA-AK149641 regulates the secretion of tumor necrosis factor-α in P815 mast cells by targeting the nuclear factor-kappa B signaling pathway
Source: Sci Rep. 2020 Oct 6;10:16655. doi: 10.1038/s41598-020-73186-x (PMC7538977; doi:10.1038/s41598-020-73186-x)
Supplement: Supplementary file 1 — Supplementary Information. [file 41598_2020_73186_MOESM1_ESM.doc]

**LncRNA-AK149641 Regulates the Secretion of Tumor Necrosis Factor-α in P815 Mast Cells by Targeting the Nuclear Factor-Kappa B Signaling Pathway**

**Authors:** Yao Zhou^1^, Li-na Gu^2^, Jie Zhang^3^, Jing Pan^3^, Jia-min Zhang^1^, De-yu Zhao^1*^, Feng Liu^1*^

^1^Department of Respiratory Medicine, Children’s Hospital of Nanjing Medical University, Nanjing, Jiangsu, China, 210008

^2^Wuxi Children’s Hospital, Wuxi, Jiangsu, China, 214000

^3^Department of Emergency Medicine, Children’s Hospital of Nanjing Medical University, Nanjing, Jiangsu, China, 210008

Yao Zhou and Li-na Gu contributed equally to this work.

**Corresponding authors**

*De-yu Zhao

Department of Respiratory Medicine

Children’s Hospital of Nanjing Medical University

No 72 Guang Zhou Road

Nanjing, Jiangsu, China

Tel: +86 18951769559

E-mail: [zhaodeyu98@126.com](mailto:zhaodeyu98@126.com)

*Feng Liu

Department of Respiratory Medicine

Children’s Hospital of Nanjing Medical University

No 72 Guang Zhou Road

Nanjing, Jiangsu, China

Tel: +86 13851718903

E-mail: [axsliu@163.com](mailto:axsliu@163.com)

**Supplementary Table1. The sequences of siRNAs for specific amplification of the 14 candidate lncRNAs**

| uc007qxz.1 | siRNA-1 | Forward: 5’-GCUCCUCAUCAUGACAUAUTT-3’ | Reverse: 5’-AUAUGUCAUGAUGAGGAGCTT-3’ |
| --- | --- | --- | --- |
|  | siRNA-2 | Forward: 5’-CCCAGCAUUUGUCUCUCAATT -3’ | Reverse: 5’-UUGAGAGACAAAUGCUGGGTT-3’ |
|  | siRNA-3 | Forward:5’-GGUAAAUUCUGAUGCUGUUTT-3’ | Reverse: 5’-AACAGCAUCAGAAUUUACCTT-3’ |
| AK033755 | siRNA-1 | Forward: 5’-GCUGUUGCCUGCAGAUGUATT-3’ | Reverse: 5’-UACAUCUGCAGGCAACAGCTT-3’ |
|  | siRNA-2 | Forward: 5’-GGCAGGCUACUUACUUUAATT-3’ | Reverse: 5’-UUAAAGUAAGUAGCCUGCCTT-3’ |
|  | siRNA-3 | Forward: 5’-CCACAUGGCCUUCAGAGAUTT-3’ | Reverse: 5’-AUCUCUGAAGGCCAUGUGGTT-3’ |
| ENSMUST00000122349 | siRNA-1 | Forward: 5’-GCAACGGUGUUAUUCAGAATT-3’ | Reverse: 5’-UUCUGAAUAACACCGUUGCTT-3’ |
|  | siRNA-2 | Forward:5’-GCGGAUGAAGAUGGGUGUUTT-3’ | Reverse: 5’-AACACCCAUCUUCAUCCGCTT-3’ |
|  | siRNA-3 | Forward: 5’-GCUCUGAUUCAGCAAGCCATT-3’ | Reverse: 5’-UGGCUUGCUGAAUCAGAGCTT-3’ |
| AK156004 | siRNA-1 | Forward: 5’-GCCAGACUUGUUGAUGCAUTT-3’ | Reverse: 5’-AUGCAUCAACAAGUCUGGCTT-3’ |
|  | siRNA-2 | Forward:5’-GGUCACAUAGAAGGAACAATT-3’ | Reverse: 5’-UUGUUCCUUCUAUGUGACCTT-3’ |
|  | siRNA-3 | Forward: 5’-GGGUAUGUAUCAUCCUUUATT-3’ | Reverse: 5’-UAAAGGAUGAUACAUACCCTT-3’ |
| AK019612 | siRNA-1 | Forward: 5’-CCCAAGUCAGCUAUGCCUUTT-3’ | Reverse: 5’-AAGGCAUAGCUGACUUGGGTT-3’ |
|  | siRNA-2 | Forward:5’-GCAGCGGGAGACAAUGUUATT-3’ | Reverse: 5’-UAACAUUGUCUCCCGCUGCTT-3’ |
|  | siRNA-3 | Forward:5’-GGUCAUAGGAAUGAAAGUUTT-3’ | Reverse: 5’-AACUUUCAUUCCUAUGACCTT-3’ |
| AK007111 | siRNA-1 | Forward: 5’-GGAACAUGCUCAGCAGAUUTT-3’ | Reverse: 5’-AAUCUGCUGAGCAUGUUCCTT-3’ |
|  | siRNA-2 | Forward:5’-GGUCACAUAGAAGGAACAATT-3’ | Reverse: 5’-AUUCGGAGGAACAUGAACCTT-3’ |
|  | siRNA-3 | Forward: 5’-GGAAACACUGAGGCUGUUATT-3’ | Reverse: 5’-UAACAGCCUCAGUGUUUCCTT-3’ |
| AK149641 | siRNA-1 | Forward:5’-GGUUUGACAGUAGCUAGUUTT-3’ | Reverse: 5’-AACUAGCUACUGUCAAACCTT-3’ |
|  | siRNA-2 | Forward:5’-CCAGCAGAGACAUCGCUAUTT-3’ | Reverse: 5’-AUAGCGAUGUCUCUGCUGGTT-3’ |
|  | siRNA-3 | Forward:5’-GCAGAUUAGAUGCUGAAUUTT-3’ | Reverse: 5’-AAUUCAGCAUCUAAUCUGCTT-3’ |
| ENSMUST00000142569 | siRNA-1 | Forward:5’-GCAGCCAGUGAAUAGGGUUTT-3’ | Reverse: 5’-AACCCUAUUCACUGGCUGCTT-3’ |
|  | siRNA-2 | Forward:5’-CCUUGCUGCCACUGAGAAUTT-3’ | Reverse: 5’-AUUCUCAGUGGCAGCAAGGTT-3’ |
|  | siRNA-3 | Forward:5’-CCUGGUGAUUGACACCUUATT-3’ | Reverse: 5’-UAAGGUGUCAAUCACCAGGTT-3’ |
| AK006687 | siRNA-1 | Forward:5’-GCCUUCUGGGAACUGUAGUTT-3’ | Reverse: 5’-ACUACAGUUCCCAGAAGGCTT-3’ |
|  | siRNA-2 | Forward:5’-GGCAUCUAUGUGGUGAUCUTT-3’ | Reverse: 5’-AGAUCACCACAUAGAUGCCTT-3’ |
|  | siRNA-3 | Forward:5’-GCCUUAACUAUCCUGCUUATT-3’ | Reverse: 5’-UAAGCAGGAUAGUUAAGGCTT-3’ |
| ENSMUST00000119808 | siRNA-1 | Forward:5’-GCGUGAGACCAUGGAAACATT-3’ | Reverse: 5’-UGUUUCCAUGGUCUCACGCTT-3’ |
|  | siRNA-2 | Forward:5’-CCCAGUGAUUUCUGGACAUTT-3’ | Reverse: 5’-AUGUCCAGAAAUCACUGGGTT-3’ |
|  | siRNA-3 | Forward:5’-UCUGUCAUGGAAGACUGUUTT-3’ | Reverse: 5’-AACAGUCUUCCAUGACAGATT-3’ |
| AK080622 | siRNA-1 | Forward:5’-CCUCCAUGAGCUAGGGAAATT-3’ | Reverse: 5’-UUUCCCUAGCUCAUGGAGGTT-3’ |
|  | siRNA-2 | Forward:5’-GGCGGAAUUCAACUUGGAUTT-3’ | Reverse: 5’-AUCCAAGUUGAAUUCCGCCTT-3’ |
|  | siRNA-3 | Forward:5’-GCAGACCGAACUCACACAUTT-3’ | Reverse: 5’-AUGUGUGAGUUCGGUCUGCTT-3’ |
| AK015424 | siRNA-1 | Forward:5’-GCUUAAACUUCAAGCCUCATT-3’ | Reverse: 5’-UGAGGCUUGAAGUUUAAGCTT-3’ |
|  | siRNA-2 | Forward:5’-GCAGAAAUCCAGAGGGCAATT-3’ | Reverse: 5’-UUGCCCUCUGGAUUUCUGCTT-3’ |
|  | siRNA-3 | Forward:5’-GGUCUUGGGCAGCUUACUATT-3’ | Reverse: 5’-UAGUAAGCUGCCCAAGACCTT-3’ |
| ENSMUST00000117724 | siRNA-1 | Forward:5’-GCAAGUAGACAGCAGUUAATT-3’ | Reverse: 5’-UUAACUGCUGUCUACUUGCTT-3’ |
|  | siRNA-2 | Forward:5’-GGAACAGCUGAUUCUGAAUTT-3’ | Reverse: 5’-AUUCAGAAUCAGCUGUUCCTT-3’ |
|  | siRNA-3 | Forward:5’-GGUCCCAGUGAUAGGGUUUTT-3’ | Reverse: 5’-AAACCCUAUCACUGGGACCTT-3’ |
| ENSMUST00000119519 | siRNA-1 | Forward:5’-UCAUCCUGUGUUGAGACUUTT-3’ | Reverse: 5’-AAGUCUCAACACAGGAUGATT-3’ |
|  | siRNA-2 | Forward:5’-GGGUUUGCUGUGCUGAAAUTT-3’ | Reverse: 5’-AUUUCAGCACAGCAAACCCTT-3’ |
|  | siRNA-3 | Forward:5’-CCUUCGGAUAAAUGUAGUATT-3’ | Reverse: 5’-UACUACAUUUAUCCGAAGGTT-3’ |
|  | siRNA-NC | Forward:5’-UUCUCCGAACGUGUCACGUTT-3’ | Reverse: 5’-ACGUGACACGUUCGGAGAATT-3’ |

**Supplementary Table2. The sequences of the primers used for lncRNA amplification**

| uc007qxz.1 | Forward: 5’-GCAGGGAAACAGAGCCATC-3’ | Reverse: 5’-AGAGACAAATGCTGGGAGGA-3’ |
| --- | --- | --- |
| AK033755 | Forward: 5’-TCTGCCCGAAATCTACTGCT-3’ | Reverse: 5’-TGTGGTGAGGCTACATCTGC-3’ |
| AK080622 | Forward: 5’-CTTCAGTGTGGCGTCCTTCT-3’ | Reverse: 5’-TGAGGTACCCAGAGCCAGG-3’ |
| ENSMUST00000122349 | Forward: 5’-AGGTCACACTGGGCTTCTGT-3’ | Reverse: 5’-AACTCATCCTTCTGGGCTTG-3’ |
| AK156004 | Forward: 5’-ACAAAACACCTACCACCACATCAA-3’ | Reverse: 5’-TCTGTGTAGCCCTGGCTGTC-3’ |
| AK019612 | Forward: 5’-AAAGCTGCCATCCCATGTCC-3’ | Reverse: 5’-CATGAACCTTCTGAGGGGAGAC-3’ |
| AK007111 | Forward: 5’-GATAATGCCGTGGGATGTG-3’ | Reverse: 5’-GGCTGGACCTTTCTGTCTTC-3’ |
| AK149641 | Forward:5’-GATGCTCTGGAACTGGAGGT-3’ | Reverse: 5’-GCGATGTCTCTGCTGGAAG-3’ |
| ENSMUST00000142569 | Forward:5’-TTCACCTGCCTCTACCTCCT-3’ | Reverse: 5’-CTTCCTGTTTGCTTCACACG-3’ |
| AK006687 | Forward:5’-CGCTAGGATGCTAAGCCGAG-3’ | Reverse: 5’-CGCTAGGATGCTAAGCCGAG-3’ |
| ENSMUST00000119808 | Forward:5’-CGCTAGGATGCTAAGCCGAG-3’ | Reverse: 5’-GATTTGGCCCTTGAATTGTT-3’ |
| ENSMUST00000119519 | Forward:5’-GATTTGGCCCTTGAATTGTT-3’ | Reverse: 5’-GATTTGGCCCTTGAATTGTT-3’ |
| AK015424 | Forward:5’-GATTTGGCCCTTGAATTGTT-3’ | Reverse: 5’-TAATCACACCTGCCCTTGCC-3’ |
| ENSMUST00000117724 | Forward:5’-TAGCTCTGGACCTGATTTTATGCT-3’ | Reverse: 5’-AGCACACTGTACCCAAGTTCC-3’ |
| GAPDH | Forward:5’-GGTTGTCTCCTGCGACTTCA-3’ | Reverse: 5’-TGGTCCAGGGTTTCTTACTCC-3’ |

**Supplementary Table3. The sequences of the primers of lncRNA-AK149641 and anti-sense lncRNA-AK149641**

| lncRNA-AK149641 | Forward:5’-GATGCTCTGGAACTGGAGGT-3’ | Reverse: 5’-GCGATGTCTCTGCTGGAAG-3’ |
| --- | --- | --- |
| Anti-sense lncRNA-AK149641 | Forward:5’-ACCTCCAGTTCCAGAGCATC-3’ | Reverse: 5’-CTTCCAGCAGAGACATCGC-3’ |

**Supplementary Table4. The sequences of primers of lncRNA-AK149641 and fmr1os.**

| lncRNA-AK149641 | Forward:5’-GATGCTCTGGAACTGGAGGT-3’ | Reverse: 5’-GCGATGTCTCTGCTGGAAG-3’ |
| --- | --- | --- |
| fmr1os | Forward:5’- ACCTGTTTGAACTGCAAGTC -3’ | Reverse: 5’- GTAGTTATCACTGTTCCTAG -3’ |
